# A performance comparison of machine learning models for stock market prediction with novel investment strategy

**DOI:** 10.1371/journal.pone.0286362

**Published:** 2023-09-21

**Authors:** Azaz Hassan Khan, Abdullah Shah, Abbas Ali, Rabia Shahid, Zaka Ullah Zahid, Malik Umar Sharif, Tariqullah Jan, Mohammad Haseeb Zafar

**Affiliations:** 1 Department of Electrical Engineering and Computer Science, Jalozai Campus, University of Engineering and Technology, Peshawar, Pakistan; 2 Department of Electrical Engineering, Main Campus, University of Engineering and Technology, Peshawar, Pakistan; 3 Cardiff School of Technologies, Cardiff Metropolitan University, Cardiff, United Kingdom; University College Dublin, PAKISTAN

## Abstract

Stock market forecasting is one of the most challenging problems in today’s financial markets. According to the efficient market hypothesis, it is almost impossible to predict the stock market with 100% accuracy. However, Machine Learning (ML) methods can improve stock market predictions to some extent. In this paper, a novel strategy is proposed to improve the prediction efficiency of ML models for financial markets. Nine ML models are used to predict the direction of the stock market. First, these models are trained and validated using the traditional methodology on a historic data captured over a 1-day time frame. Then, the models are trained using the proposed methodology. Following the traditional methodology, Logistic Regression achieved the highest accuracy of 85.51% followed by XG Boost and Random Forest. With the proposed strategy, the Random Forest model achieved the highest accuracy of 91.27% followed by XG Boost, ADA Boost and ANN. In the later part of the paper, it is shown that only classification report is not sufficient to validate the performance of ML model for stock market prediction. A simulation model of the financial market is used in order to evaluate the risk, maximum draw down and returns associate with each ML model. The overall results demonstrated that the proposed strategy not only improves the stock market returns but also reduces the risks associated with each ML model.

## Introduction

Stock markets being one of the essential pillars of the economy have been extensively studied and researched [1]. Forecasting the stock price is an essential objective in the stock market since the higher expected return to the investors can be guaranteed with better prediction [2]. The price and uncertainty in the stock market is predicted by exploiting the patterns found in the past data [3]. The nature of the stock market has always been vague for investors because predicting the performance of a stock market is very challenging. Various factors like the political disturbance, natural catastrophes, international events and much more must be considered in predicting the stock market [4]. The challenge is so huge that even a small improvement in stock market prediction can lead to huge returns.

The stock market can only move in one of the two directions: upwards (when stock prices rise) or downwards (when stock prices fall) [5]. Generally, there are four ways to analyze the stock market direction [6]. The most basic type of analysis is the fundamental analysis, which is the way of analyzing the stock market by looking at the company’s economic conditions, reports and future projects [7]. The second and most common technique is technical analysis [8]. In this method, the direction of the stock market is anticipated by looking at the stock market price charts and comparing it with its previous prices [9]. The third and most advanced technique is the Machine learning (ML) based analysis that analyzes the market with less human interaction [10]. ML models find the patterns inside historical data based on which they try to forecast the stock market prices for the future. The fourth technique, called sentimental-based analysis, analyzes the stock market prices by the sentiments of other individuals like activity on social media or financial news websites [11].

The difficulty of the stock market prediction drew the attention of numerous researchers worldwide. A number of papers have been presented that could predict the stock prices based on ML models. These models include Artificial Neural Network (ANN) [12], Decision Tree (DT) [13], Support Vector Machine (SVM) [14], K-Nearest Neighbors (KNN) [15], Random Forest (RF) [16] and Long Short-Term Memory networks (LSTM) [17]. The proposed systems either used a single ML model optimized for specific stocks [18–20], or multiple ML models in order to analyze their performance on different stocks [21–24]. Many advanced techniques like hybrid models were also employed in order to improve prediction accuracy [25–27].

Different ML models like RF and stochastic gradient boosting were used to predict the prices of Gold and Silver with an accuracy of more than 85% [18]. A novel model based on SVM and Genetic Algorithm, called Genetic Algorithm Support Vector Machine (GASVM), was proposed to forecast the direction of Ghana Stock Exchange [19]. The proposed model achieved an accuracy of 93.7% for a 10-day stock price movement outperforming other traditional ML models. The Artificial Neural Network Garch (ANNG) model was used to forecast the uncertainty in oil prices [20]. In this model, first, the GARCH model is used to predict the oil price. This prediction is then used as input to ANN for improvement in the overall commodity price forecast by 30%.

Different ML models perform differently on the same historical data. Their performance depends on the type of data and the duration for which the past data is available. In many recent papers, multiple ML models were used on the same financial time series data to predict the future price of the stock to see the performance of each ML model [21–24]. Comparative analysis of nine ML and two Deep Learning (DL) models was performed on Tehran stock market [21]. The main purpose of this analysis was to compare the accuracy of different models on continuous and binary datasets. The binary dataset was found to increase the accuracy of models. In [22], four ML models (ANN, SVM, Subsequent Artificial Neural Network (SANN) and LSTM) were used to predict the Bitcoin prices using different time frames. The results show that SANN was able to predict the Bitcoin prices with an accuracy of 65%, whereas LSTM showed an accuracy of 53% only. In another comparative study [23], four ML models (Multi-Layer Perceptron (MLP), SVM and RF) were used to forecast the prices for different crypto-currencies like Bitcoin, Ethereum, Ripple and Litecoin using their historical prices. MLP outperformed all other models with an accuracy ranging from 64 to 72%. Similar study was performed in [24] showing the performance comparison of different ML models on the same data.

In some recent studies, hybrid models (a combination of different ML models) are used to forecast stock prices. A hybrid model designed with the SVM and sentimental-based technique was proposed for Shanghai Stock Exchange prediction [25]. This hybrid model was able to achieve the accuracy of 89.93%. A system consisting of k-mean clustering and ensemble learning technique was developed to predict the Chinese stock market [26]. The hybrid prediction model obtained the best forecasting accuracy of the stock price on Chinese stock market. Another hybrid framework was developed in [27] for the Indian Stock Market, this model was developed using SVM with different kernel functions and KNN to predict profit or loss. The proposed system was used to predict the future of stock value. Although the accuracy of the hybrid systems is much higher but they are too complex to be implemented in real-life. Furthermore, a comparative analysis of the prior and proposed study has been shown in Table 1.

**Table 1:** Comparative analysis of previous and proposed study.

Ref	Contribution	Results	Limitation of Current Literature and Proposed study
[16]	Four trading strategies based on a random forest classifier to predict S&P500.	The best performance accuracy, 44.78%, is accomplished with the De Luca and Termini	In almost all the proposed ML-based systems, the performance of the ML models were only gauged by their classification ability. It is insufficient to determine the performance of the ML model for stock market prediction. The classification metrics do not take into the account some important fact ors like returns, maximum draw down, risk-to-reward ratio, transactional cost and the risks associated with each ML model. In this study ML models are compared on the basis of both Classific ation as well as financial metrics which makes this work more valuable as compare to the current literature.
[18]	RF and stochastic gradient boosting were used to predict the prices of Gold and Silver.	Achieved an accuracy of 85%.	
[19]	GASVM was proposed to forecast the direction of Ghana Stock Exchange	Achieved an accuracy of 93.7%.	
[20]	ANNG model was used to forecast the uncertainty in oil prices.	Improve commodity price prediction by 30%.	
[21]	Comparison of Nine ML and two DL models was performed on Tehran stock market.	DL model outperformed other models with an accuracy of 86%	
[22]	ANN, SVM, SANN and LSTM were used to predict the Bitcoin prices.	SANN model outperformed other models with an accuracy of 65%.	
[23]	MLP, SVM and RF were used to forecast the prices for different crypto-currencies.	MLP outperformed all other models with an accuracy of 64 to 72%.	
[24]	A novel ensemble machine learning framework was proposed to predict the Chinese stock market.	Accuracy of more than 60% was claimed for some trend patterns.	

In almost all the proposed ML-based systems, a primary limitation has been observed in the empirical results. The performance of the ML models were only gauged by their classification ability. Although, it is one of the important parameters being used for the evaluation of the ML model, but it is insufficient to determine the performance of the ML model for stock market prediction. The classification metrics do not take into the account some important factors like returns, maximum draw down, risk-to-reward ratio, transactional cost and the risks associated with each ML model. These factors must be considered in the evaluation of ML models for stock market predictions.

### Research cContributions

The following are the major contributions of paper:

- A performance comparison of nine ML models trained using the traditional methodology for stock market prediction using both performance metrics and financial system simulations.
- Proposing a novel strategy to train the ML models for financial markets that perform much better than the traditional methodologies.
- Proposing a novel financial system simulation that provides financial performance metrics like returns, maximum drawdown and risk-to-reward ratio for each ML model.

### Paper organization

The rest of the paper is organized as follows: The next section explains the proposed methodology used in training nine ML models for stock market prediction. Section III analyses the outcomes of simulation models in detail. This section consists of ML models simulation as well as Financial models simulations. The conclusions and future directions are discussed in Sections IV and V respectively.

## Methodology

In this paper, a software approach is used to apply different ML algorithms to predict the direction of the stock market for Tesla Inc. [28]. This prediction system is implemented in Python using frameworks like Scikit-learn [29], Pandas [30], NumPy [31], Alpaca broker [32] and Plotly [33].

**Fig 1:**
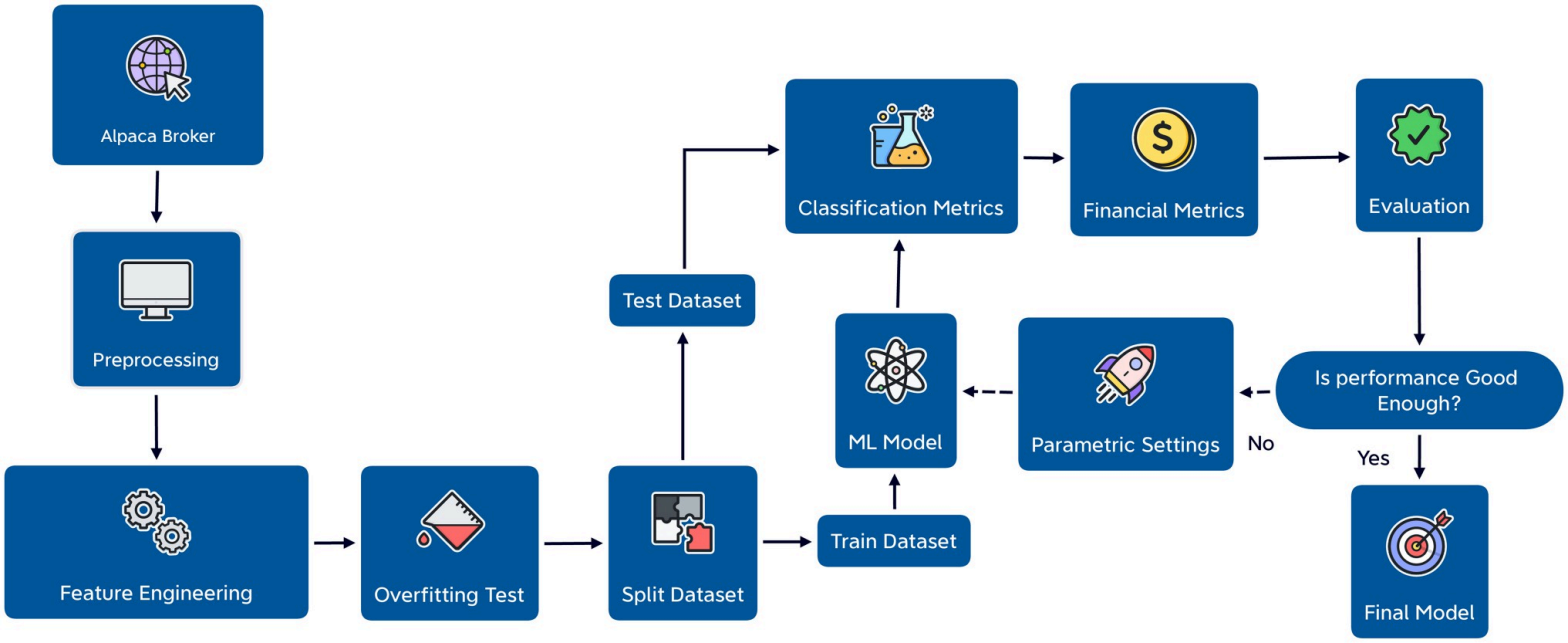
Flow chart of the proposed prediction system.

The flowchart of the methodology is illustrated in Fig 1. The first step is to import the stock market data from Alpaca broker and preprocess it using various techniques. The imported stock market data has some information that is not needed in the proposed system. This unwanted data, like trade counts and volume-weighted average price, is removed in the preprocessing stage. Preprocessing also involves handling missing stock prices and cleaning data from unnecessary noise. Missing values can be estimated using interpolation techniques or just by taking the mean value of the point before and after the missing point.

Traditionally, the stock price at the end of the day (EOD) is used in ML-based systems. The variation in the stock price is usually the most in the first hour after the market is open. So, stock price within this hour is more effective than the EOD stock price. The direction of the market is set by the business done in this hour. So, in this paper, the stock price after 15 minutes, when the stock market is open, will also be extracted. The results from the stock price at EOD will be compared with the results from the proposed 15 minutes strategy.

Once the stock price data has been extracted, the subsequent stage involves computing various input features from the technical indicators and statistical formulas. Nine input features, listed in Table 2, are selected for the prediction purposes. These calculated input features are subjected to overfitting tests. These tests are essential because overfit data can cause reduction in the accuracy of the ML models [34].

**Table 2:** Selected input feature variables for ML models.

Feature Variables	Time Period
RSI [24]	14
SMA [24]	50
ADX [24]	20
Volume	n/a
Correlation	24
Previous (Open–Close)	n/a
Previous (Close–High)	n/a
Previous (Close–Low)	n/a
Momentum	20

RSI = Relative Strength Index, SMA = Simple Moving Average, ADX = Average Directional Movement Index

Following the overfitting tests, the input data is divided into training and testing data. The data is then normalized using Min-Max normalization technique to prevent the biasing phenomenon. Normalization is performed using the following Eq (1):

$$\begin{array}{c} X_{norm}=\frac{X-X_{min}}{X_{max}-X_{min}} \end{array}$$ (1)

The input features and output variables are provided to the ML models in order to detect the patterns within the training data. Various ML models have been employed in this study. Table 3 shows the selected nine ML models to predict the direction of the stock market in this paper. The optimal parameters for each ML models are selected through GridSearchCV [35]. A scikit-learn function that helps in selecting best performing parameter for a particular model. After choosing the optimal parameters, the ML models are trained and tested.

**Table 3:** Selected ML models for stock market prediction.

ML models	Reference
Support Vector Machine (SVM)	[36]
Decision Tree (DT)	[37]
Logistic Regression (LR)	[38]
Naive Bayes (NB)	[39]
K Nearest Neighbor (KNN)	[40]
Random Forest (RF)	[41]
Adaptive Boosting (ADA BOOST)	[42]
Extreme Gradient Boosting (XG BOOST)	[43]
Artificial Neural Network (ANN)	[44]

In the next step, the outcome of the trained ML models is assessed using some performance metrics. There are a number of classification metrics that can be used to evaluate the performance of an ML algorithm [45]. Usually, three most powerful measures are chosen to classify these models with respect to their performance. The measures are accuracy, F1 score and Receiver Operator Characteristic and Area Under the Curve (ROC_AUC) [46]. The equations for Accuracy and F1_score are shown below:

$$\begin{array}{c} Accuracy=\frac{TP+TN}{TP+TN+FN+FP} \end{array}$$ (2)

$$\begin{array}{c} F1\_score=\frac{2*(Precision*Recall)}{\left(Precision+Recall\right)} \end{array}$$ (3)

For evaluation purposes the accuracy, ROC_AUC and F1_score are useful measures, however, they are not sufficient for all problems. Recall and precision are two additional well-known metrics for classification problems [47, 48]. The expression for Recall and Precision are also shown in below:

$$\begin{array}{c} Recall=\frac{TP}{TP+FN} \end{array}$$ (4)

$$\begin{array}{c} Precision=\frac{TP}{TP+FP} \end{array}$$ (5)

Additionally, a confusion matrix is used to summarize the performance of each ML model. It provides detailed insight into ML predictions by indicating False Positives (FP), True Positives (TP), False Negatives (FN) and True Negatives (TN) [49]. False Positives show that the model prediction is true while the real sample is false; True Positives show that the model prediction and the real sample both are true; False Negatives represent that the model prediction is false while the real sample is true; True Negatives show that the model prediction and real sample both are false.

In the next step, a novel financial model is developed and simulated to analyze the performance of the trained ML models. The financial performance metrics like Sharpe ratio, maximum drawdown, cumulative return and annual return [50] are used to analyze the performance of the trained ML models.

The Sharpe ratio is the measure of risk-free return while the maximum drawdown is the greatest decline in the value of the portfolio [51]. The equations for Sharpe ratio and maximum drawdown are shown in below:

$$\begin{array}{c} Sharpe\;ratio=\frac{\left(R_{p}-R_{f}\right)}{\sigma } \end{array}$$ (6)

$$\begin{array}{c} Maximum\;drawdown=\frac{\left(P-L\right)}{P} \end{array}$$ (7)

where R_p_ = Return of portfolio, R_f_ = Risk free rate, *σ* = Std of portfolio excess return, P = Peak value before largest drop, and L = Lowest value before new high.

Annual return is the return gained during the period of one year while the cumulative return is the total return on the invested capital within any specific time frame. The expressions for annual return and cumulative return are shown in Eqs (8) and (9).

$$\begin{array}{c} Annual\;return={\left(\frac{E}{I}\right)}^{1/n}-1 \end{array}$$ (8)

$$\begin{array}{c} Cumulative\;return=\frac{E-I}{100} \end{array}$$ (9)

where, E = Ending value, I = Initial value and n = Number of years.

## Experimental results

### Dataset description and project specifications

Tesla Inc. is a major American automobile company producing technologically advanced electric vehicles. The company has recently obtained a lot of attention due to its stock prices. A drastic increase in revenue in the year 2021 made Tesla stocks very appealing for capitalists and investors around the world as shown in Table 4 [52].

**Table 4:** Annual growth of Tesla Inc. stocks.

Stock Company	Year	Annual Growth (%)
Tesla Inc	2016	73.00
	2017	68.00
	2018	82.50
	2019	14.52
	2020	28.31
	2021	70.67

Table 4 shows the annual growth of Tesla from 2016 to 2021. There has been an increase of almost 70.67% in the year 2021. By taking into account the stock volatility in the previous years and its recent growth, Tesla Inc. is an ideal candidate for this study.

**Fig 2:**
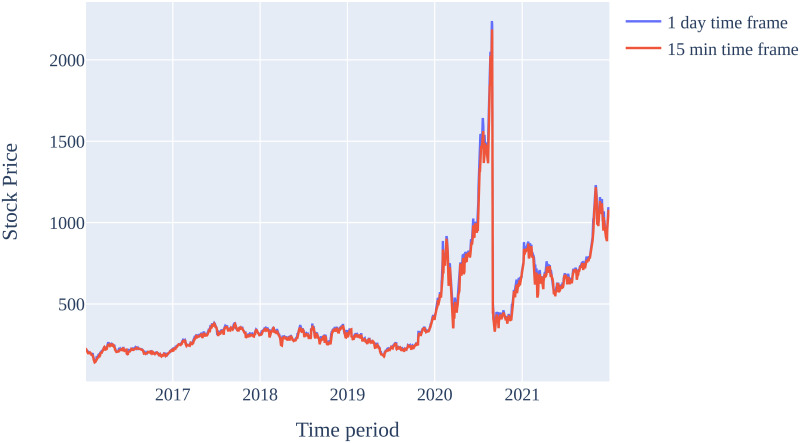
Imported stock prices of Tesla Inc. from Alpaca broker.

The stock prices for Tesla Inc. from 2016 to 2021 are considered for experimental evaluations in this paper. Furthermore, the data is split into training data and test datasets. Table 5 shows the ranges of our datasets. The stock market data for Tesla Inc., downloaded from Alpaca broker, from 2016 to 2021 is shown in Fig 2. Additionally, the project specifications can be found in Table 6.

**Table 5:** Training data and test data ranges for Tesla Inc. stocks.

Data Ranges	Start date	End date	No. of days
Total data	2016–01–01	2021–12–31	2191 days
Training data	2016–01–01	2020–11–15	1744 days
Test data	2020–11–16	2021–12–31	410 days

**Table 6:** System specifications for proposed system.

Specifications	Components/Requirements
Accuracy	More than 90%
Scope	The proposed study is limited to the Tesla Inc. stocks
Data Sources	Historical stock market data from Alpaca Broker
Type of data	Stock market historical prices from Jan 2016 to Dec 2021
Frequency of prediction	1 Day time frame
Analysis Method	Classification Analysis, Financial Analysis
Data processing	Data cleaning, Feature selection, Data normalization, Correlation test, Stationary test
Output Format	Desktop dashboard

### Machine learning models simulation

First, the optimal parameters settings for the nine ML models are selected through GridSearchCV. The selected optimal parametric settings for each model are shown in Table 7.

**Table 7:** Optimal parametric settings for each ML model.

ML Models	Parameters	Values
SVM	C	1
	Kernel	RBF
	Degree	3
Decision Tree	Criterion	Gini
	Random State	30, 20
	Min Sample Split	3, 4
Logistic Regression	Solver	1bfgs
	Loss function	gradient descent
	Max iteration	100
Naive Bayes	C	1
	Algorithm	Gaussian
KNN	K neighbors	20, 30
	Leaf size	30,20
	Metric	Minkowski
Random Forest	N estimators	80, 100
	Criterion	Gini
	Random State	30, 20
	Min Sample Leaf	4
ADA Boost	N estimator	40, 60
	Algorithm	SAMME.R
	Learning Rate	1
XG Boost	Max Depth	7
	N estimator	40, 60
	Random State	30, 20
ANN	Activation functions	Relu, Sigmoid
	Hidden Layer Neutrons	100
	Max epochs	20
	Optimizer	Adam

The simulations for stock market prediction are performed using Python on a Jupiter notebook. ML models were evaluated using Tesla Inc. stock prices for a 1-day time frame and 15-min time interval strategy. These models were first trained on the data from Jan 01, 2016 to Nov 15, 2020. The trained models were then validated on the test data from Nov 16, 2020 to Dec 31, 2021 as shown in Table 5.

Tables 8–10 show the classification report for nine different ML models. Tables 8 and 9 show the performance metrics for different ML models for a 1-day time frame and 15-min time interval strategy. These tables list the accuracy, F1 score, ROC AUC, precision and recall in percentage for all of the ML models. Table 10 shows the confusion matrix for the ML models. It lists the number of correct and wrong predictions made by each ML model.

**Table 8:** Classification metrics for Tesla Inc. stocks for 1-day time frame data.

ML Models	Accuracy (%)	F1_score (%)	ROC_AUC (%)	Precision (%)	Recall (%)
Decision Tree	83.01	83.00	83.58	83.50	83.50
Logistic Regression	85.51	85.50	85.77	85.50	86.00
KNN	79.15	79.12	79.49	79.50	79.50
Naive Bayes	73.49	70.10	70.50	79.50	70.50
Random Forest	84.45	85.11	85.13	85.00	85.50
ADA Boost	83.74	84.53	84.97	85.00	84.00
SVM	82.68	82.51	82.82	82.50	83.00
XG Boost	84.80	85.52	85.45	85.50	85.00
ANN	84.45	84.50	90.95	84.50	84.50

**Table 9:** Classification metrics for Tesla Inc. stocks for the proposed 15-min time interval strategy.

ML Models	Accuracy (%)	F1_score (%)	ROC_AUC (%)	Precision (%)	Recall (%)
Decision Tree	88.10	88.50	88.96	88.00	88.50
Logistic Regression	90.60	90.55	90.52	90.50	90.50
KNN	80.53	80.50	80.37	81.00	80.00
Naive Bayes	81.54	81.50	81.77	82.50	81.50
Random Forest	91.27	91.00	91.28	92.00	91.50
ADA Boost	90.93	91.02	91.03	91.50	91.00
SVM	88.59	88.50	88.49	89.00	88.50
XG Boost	90.93	91.00	91.53	91.00	90.50
ANN	89.93	90.00	90.63	90.00	90.00

**Table 10:** Confusion metrics for ML models.

Prediction Models	Actual Labels	Tesla Stock 1-day		Tesla Stock 15-min	
Predict Labels		Move up	Move down	Move up	Move down
Decision Tree	Move up	124	34	127	18
	Move down	14	111	19	147
Logistic Regression	Move up	132	26	128	17
	Move down	15	110	11	142
KNN	Move up	121	37	108	37
	Move down	22	103	21	132
Naive Bayes	Move up	152	6	131	14
	Move down	69	56	41	112
Random Forest	Move up	130	28	130	15
	Move down	16	109	11	142
ADA Boost	Move up	131	27	137	8
	Move down	19	106	19	134
SVM	Move up	129	29	123	22
	Move down	20	105	12	141
XG Boost	Move up	130	28	136	9
	Move down	15	110	18	135
ANN	Move up	131	27	130	15
	Move down	17	108	15	138

#### ML models simulation results for 1-day time frame

Table 8 shows the performance metrics of nine ML models optimized for a 1-day time frame. As shown in the table, the Logistic Regression achieved the highest accuracy of 85.51% while the Naive Bayes model is found to be the least accurate model with an accuracy of 73.49%. Other classification metrics in Table 8 show a similar tendency with Logistic Regression having the best performance followed by XG Boost and Random Forest.

The confusion matrix in Table 10 shows a similar trend. For Logistic Regression, the True Positives are 132 and the False Positives are 26 for the ‘Move Up’ class. The True Negatives are 110 and the False Negatives are 15 for the ‘Move Down’ class.

Based on the discussion above, it can be seen that the performance of Logistic Regression model is better than the rest of the models for 1-day time frame. Even though its accuracy among the nine ML models is only 85.51%.

**Fig 3:**
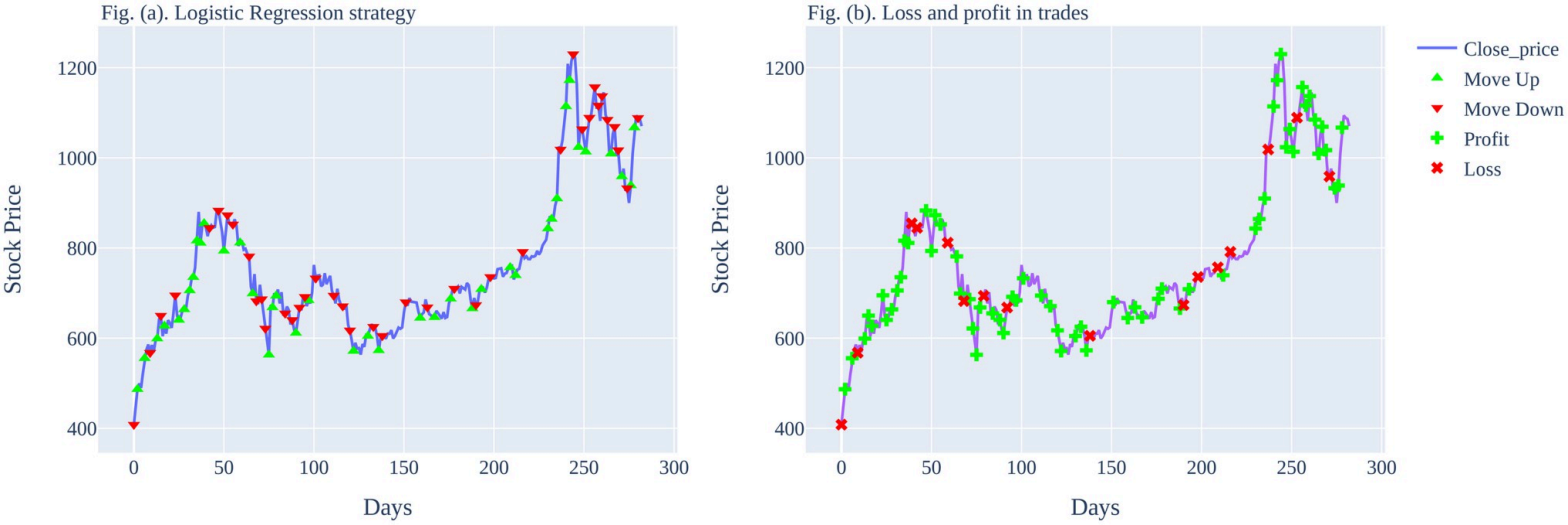
Graphical illustration of Logistic Regression predictions on Tesla stocks for (1-day time frame).

The graphical illustration of the predictions made by the Logistic Regression model for a 1-day time frame can be seen in Fig 3. It can be seen that the trained Logistic Regression model is able to make more profits than losses. However, it is interesting to note that sometimes the predictions made by the LR model are wrong in the consecutive trades that results in more drawdown. For example, during the period 180 to 230 days, there are a total of 6 trades executed, out of which 4 are losses and 2 are profitable trades.

#### ML model simulation results for the proposed 15-min strategy

In this paper, a novel 15-min time interval strategy has been proposed. In this strategy, the initial 15-min time interval is filtered out from 1-day time frame. Then the filtered 15-min time frame is used to train and validate the ML models in order to make prediction for the time frame of 1-day.

Table 9 shows the performance metrics of the ML models optimized for a 15-min time interval strategy. As shown in Table, the Random Forest achieved the highest accuracy of 91.27% followed by XG Boost and ADA Boost model. The KNN model is found to be the least accurate model with an accuracy of 80.53%. Other classification metrics in Table 9 show a similar tendency with the Random Forest having the best performance model.

The confusion matrix in Table 10 shows a similar trend. For Random Forest, the True Positives are 130 and the False Positives are 15 for the ‘Move Up’ class. The True Negatives are 142 and the False Negatives are 11 for the ‘Move Down’ class. When the results in Tables 8 and 9 are compared, it can be observed that by employing the proposed methodology, the performance of all the ML models has been greatly improved.

**Fig 4:**
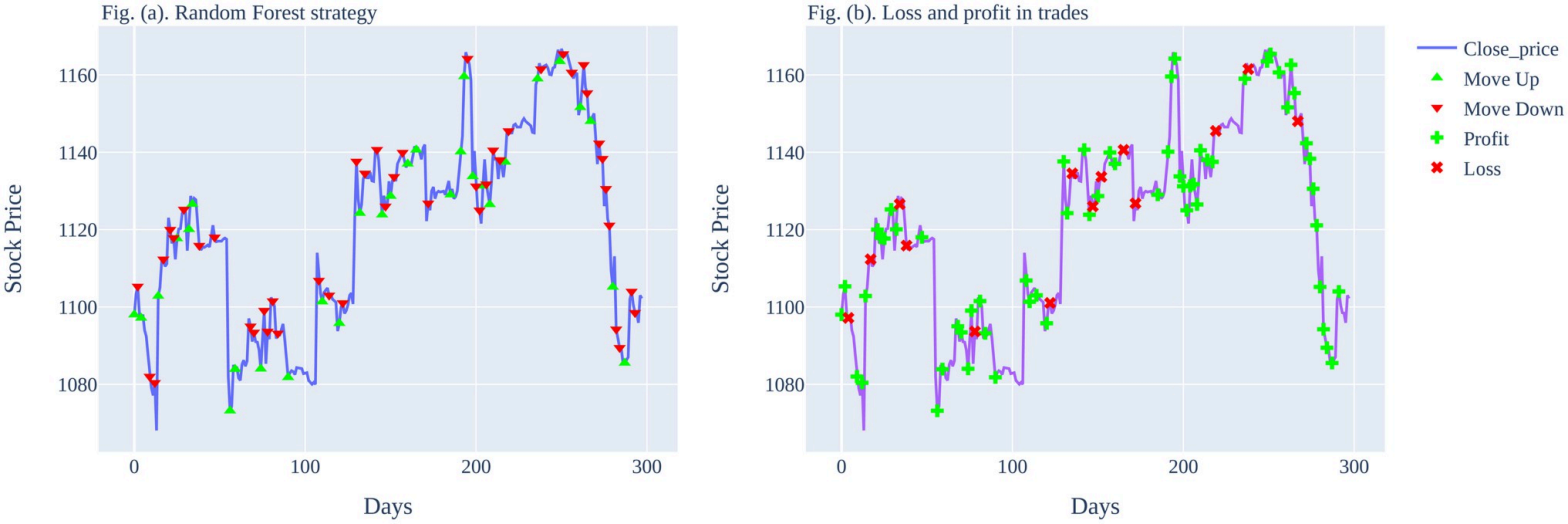
Graphical illustration of Random Forest predictions on Tesla stocks for (15-min time interval strategy).

The graphical illustration of the predictions made by the Random Forest model is shown in Fig 4, it shows the loss and profit in trades. It can also be observed that by using our proposed strategy, the number of consecutive losses has also been reduced. As shown in Fig 4(b), there are only 2 consecutive losses, which occurred during the period of 150 to 200. Factually, the proposed methodology has not only improved the performance metrics of the ML models but it also reduced the number of consecutive losses.

### Financial models simulation

In this section, a novel financial simulation model is built that is able to make investment based on the decision of the ML model. Each ML model is evaluated using financial parameters to validate their performance and suitability for real-time stock market trading. The performance of ML models is gauged using cumulative return, annual return, maximum drawdown, Sharpe ratio and capital in hand at the end of the investment period.

Initially, a USD 10k is invested. A commission fee of 0.1% (Alpaca standard commission fee) is set for each buy or sell trade. Based on the prediction by the ML model, a decision regarding buying, holding or shorting a share is taken. A single share is bought or sold on each trade to validate the performance of ML models.

**Fig 5:**
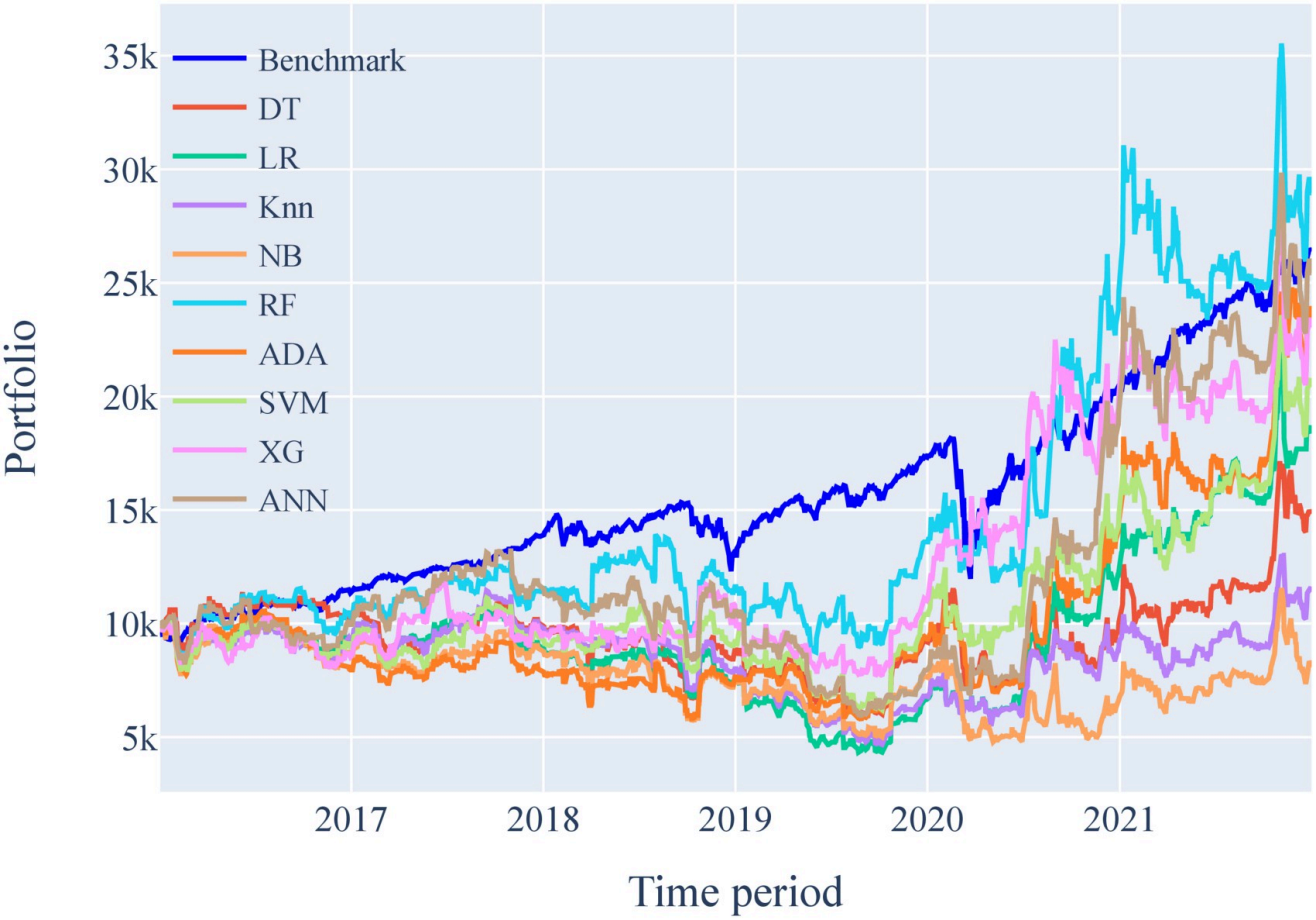
Portfolio analysis of ML models on Tesla Inc. stocks for 1-day time frame.

**Fig 6:**
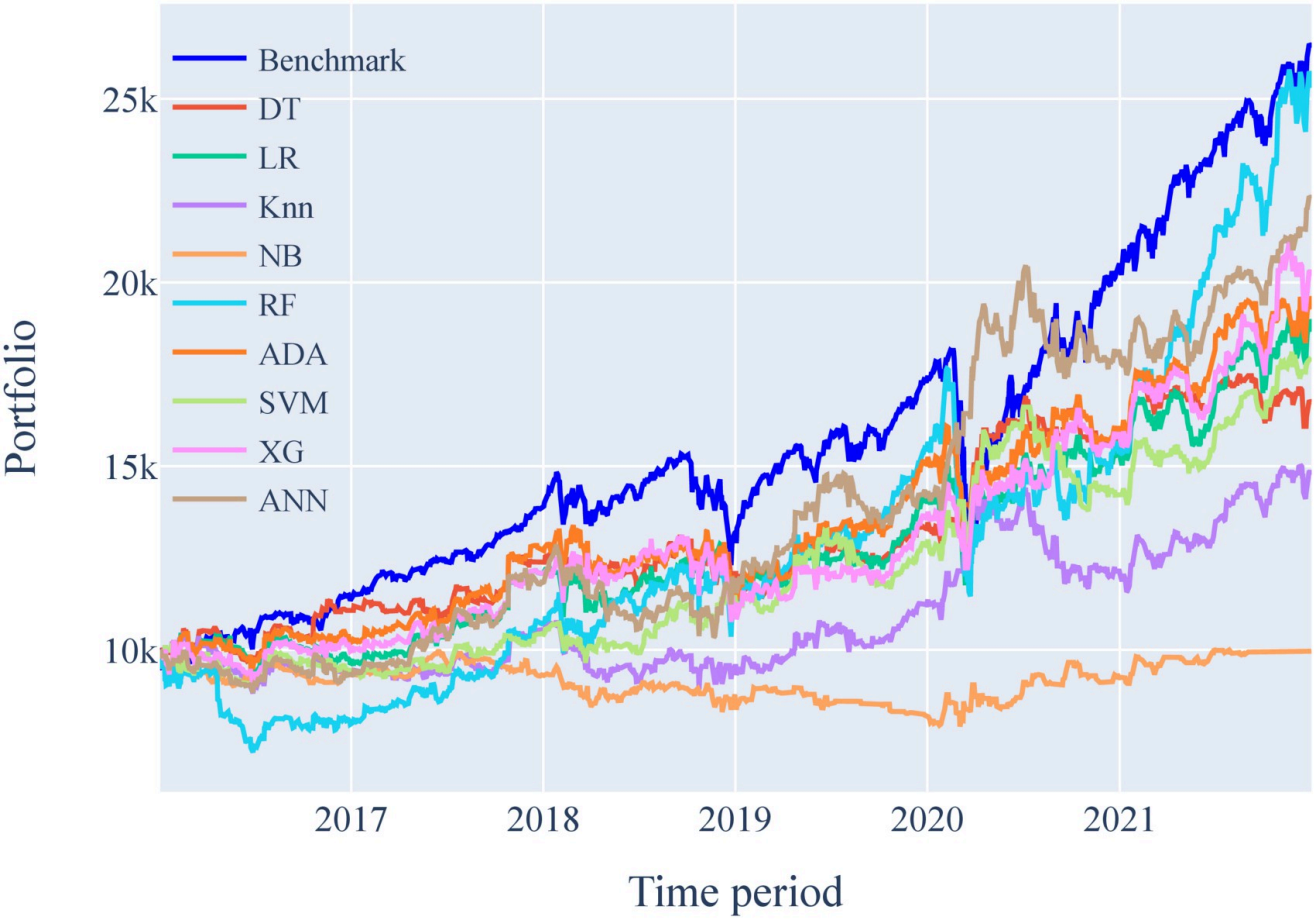
Portfolio analysis of ML models for Tesla Inc. stocks on the proposed 15-min time interval strategy.

Figs 5 and 6 show the portfolio performance of ML models on Tesla Inc. stocks for a 1-day time frame and 15-min time interval strategy. These figures show how initial capital is used to buy and sell shares based on the decision made by the ML models. Each box in the figure represents one full year from Jan 01 till Dec 31. The portfolio of each ML model is compared to a benchmark that serves as a reference for all models. This benchmark is obtained using the positive gains of stock prices.

#### Financial simulation results for 1-Day time frame data

**Fig 7:**
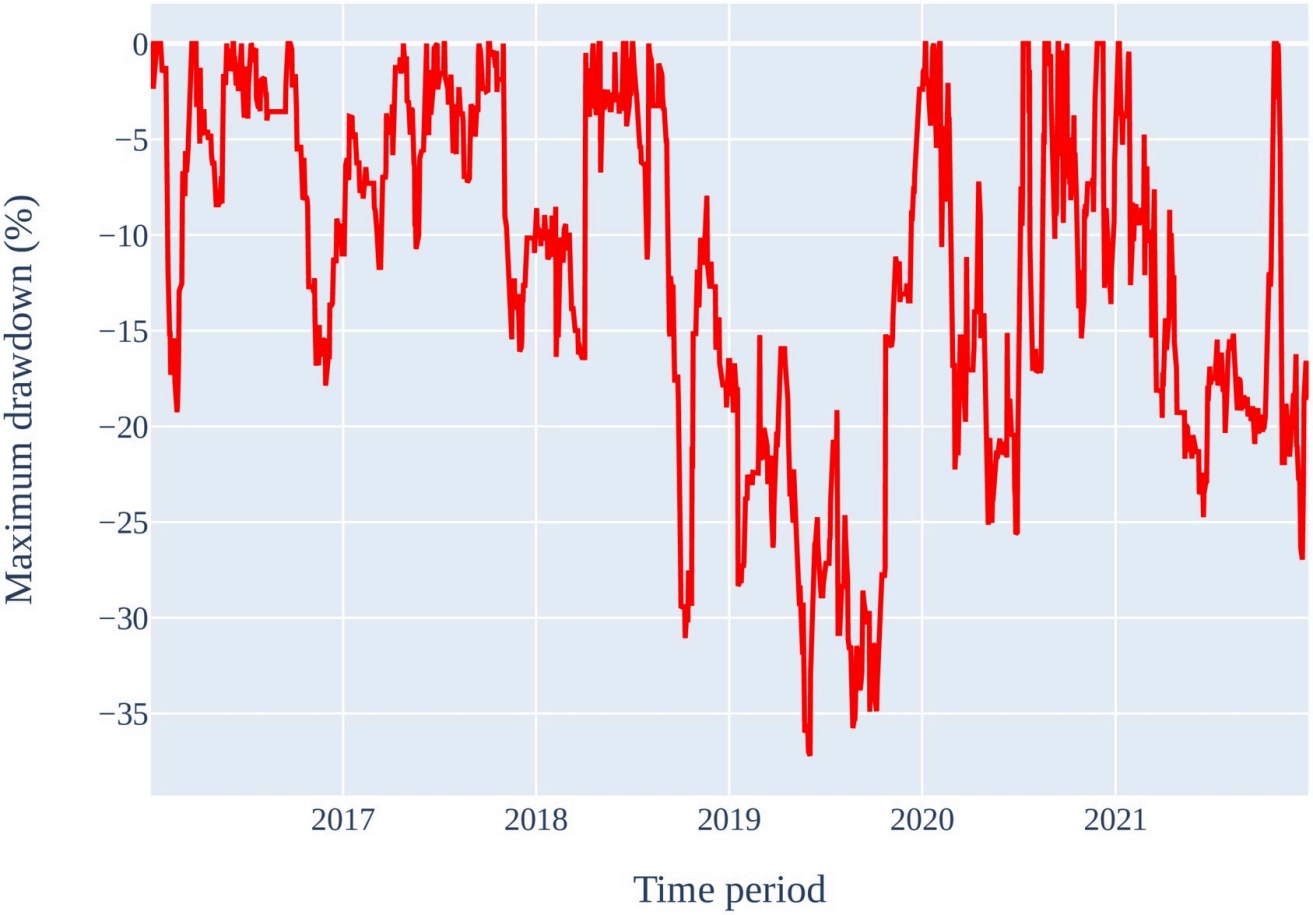
Maximum drawdown of Random Forest strategy for Tesla Inc. stocks on 1-day time frame.

The simulated outcomes of the ML models to forecast the stock price of Tesla Inc. for a 1-day period are displayed in Table 11. In the previous section, it was shown that Logistic Regression had the highest accuracy as compared to the other ML models. Therefore, it is expected that this ML model will generate highest revenue. However, the outcome of the financial simulations shows different results. It can be seen in Table 11 that the Random Forest is the best ML model with an ending capital of USD 28,966. It has a cumulative return of 189.66%, and an annual return of 19.48%, with the highest Sharpe ratio of 0.68. The Random Forest did poorly at first but after the 2019 financial market crisis, it outperformed all other ML models. The maximum drawdown of the Random Forest model is -37.21% which happened during 2019 financial crisis as shown in Fig 7. This is the lowest drawdown by any ML model.

**Table 11:** Financial performance of ML models for Tesla Inc. stocks on 1-day time frame.

Prediction Models	Cum Return (%)	Annual Return (%)	Max Draw down (%)	Sharpe Ratio	Ending Capital (USD)
Decision Tree	48.35	6.82	-48.11	0.36	14835
Logistic Regression	83.69	10.71	-59.35	0.47	18369
KNN	14.00	2.22	-59.25	0.23	11400
Naive Bayes	-19.16	-3.50	-53.85	0.10	8084
Random Forest	189.66	19.48	-37.21	0.68	28966
ADA Boost	135.91	15.44	-45.76	0.58	23591
SVM	104.10	12.69	-44.23	0.51	20417
XG Boost	130.37	14.99	-35.79	0.57	23037
ANN	154.46	16.92	-55.77	0.62	25446

The reason for better revenue generation by the Random Forest model is the quality of each True Positive and True Negative outcome. Even though the accuracy of the model is inferior to the Logistic Regression, each of its correct prediction resulted in more profit. The annual growth of Tesla Inc. from 2020 to 2021 is more than 70% as shown in Table 4. Any correct prediction during this time will result in greater revenue generation. Random Forest model outperformed all other models during this time as shown in Fig 5. Among the ML models, the Naive Bayes model shows the worst performance. Fig 5 shows that the Naive Bayes model is negative most of the time during the simulation. It is the only model with a negative cumulative return of -19.16% and worst Sharpe ratio of 0.1.

#### Financial simulation results for the proposed 15-min strategy

The portfolio performance of the ML models using the proposed approach of a 15-min time interval strategy is shown in Fig 6. This figure shows that the performance of some of the models has improved significantly when compared with a 1-day time frame. It can also be noticed that the models maintained their stability throughout the financial crisis of 2019, which indicates a significant improvement in the real-time performance of the models.

**Fig 8:**
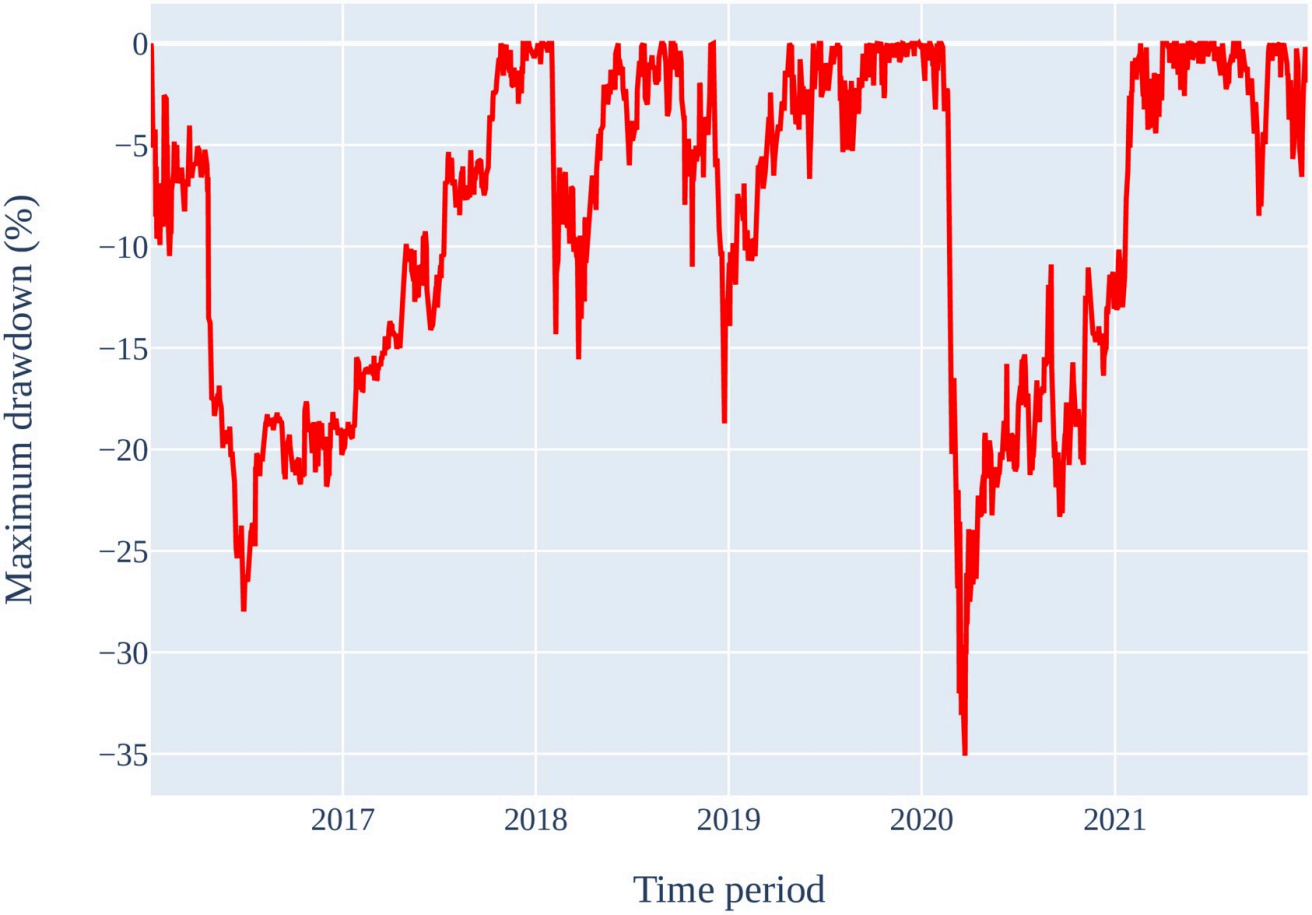
Maximum drawdown of Random Forest strategy for Tesla Inc. stock on the proposed 15-min time interval strategy.

Table 12 displays the outcome of the financial model simulation of ML models trained and validated on Tesla Inc. stocks for a 15-min time interval strategy. As expected, it can be seen that the Random Forest is the best performing model with an ending capital of USD 25,300. It records a cumulative return of 153% and annual return of 16.80% with the highest Sharpe ratio of 0.79. The maximum drawdown by the Random Forest model is—35.09% as shown in Fig 8, but it still able to generate the highest ending capital.

**Table 12:** Financial performance of ML models for Tesla Inc. stock on the proposed 15-min time interval.

Prediction Models	Cum Return (%)	Annual Return (%)	Max Draw down (%)	Sharpe Ratio	Ending Capital (USD)
Decision Tree	67.51	9.02	-12.74	0.73	16751
Logistic Regression	86.59	11.00	-21.17	0.68	18659
KNN	47.67	6.74	-19.33	0.51	14767
Naive Bayes	-0.36	-0.06	-20.85	0.05	9964
Random Forest	153	16.80	-35.09	0.79	25300
ADA Boost	92.73	11.60	-21.23	0.71	19273
SVM	79.52	10.29	-17.04	0.77	17952
XG Boost	101.77	12.46	-18.01	0.74	20177
ANN	122.94	14.36	-19.33	0.91	22294

The above discussion shows that KNN is the worst performing model on the proposed strategy. Although, Random Forest is the best model in terms of portfolio returns but ANN is the most rewarding model with a Sharpe ratio of 0.91 on the proposed 15-min time interval strategy.

## Conclusion

In this paper, nine ML models are used to predict the direction of the Tesla Inc. stock prices. The performance of this stock is first assessed for a 1-day time frame followed by a proposed 15-min time interval strategy. Following the traditional methodology, the Logistic Regression achieved the highest accuracy of 85.51% while Naive Bayes model is found to be the least accurate model with an accuracy of 73.49%. The proposed strategy significantly improved the classification performance of the ML models. With this strategy, the Random Forest model achieved the highest accuracy of 91.93% followed by XG Boost and ADA Boost. Conversely, the KNN model is found to be the least accurate model with an accuracy of 80.53%.

In this paper, it was shown that only classification metrics are not enough to justify the performance of ML models in the stock market. These metrics do not consider important factors like risk, maximum draw down and returns associate with each ML model. A simulation model of the financial market is used to simulate the trained ML models so that their performance is gauged with actual investment strategies. The evaluated results revealed that although some models are performing well in terms of portfolio returns on a traditional methodology but models on the proposed 15-min time frame strategy are significantly better in terms of risk to reward ratio and maximum drawdown. The evaluated result shows that Random Forest outperformed other models in terms of returns in both 1-day and 15-min time interval strategy.

Some other interesting observations are revealed by the comparison of the classification and financial results. The Logistic Regression model has the highest accuracy for a 1-day time frame data. So, it was expected that this ML model will generate the highest revenue. However, the outcome of the financial simulations showed different results. Similarly, the accuracy of the Random Forest model for a 15-min time interval strategy was much higher than the accuracy of the Random Forest model for a 1-day time frame. But instead of generating higher revenue on 15-min time frame strategy, it generated higher revenue on 1-day time frame. The above discussion revealed that however, the accuracy of the ML models is an important factor but the quality of each true positive outcome and true negative outcome is an equally important factor in the performance evaluation of the ML models for stock market prediction.

The overall results show that the proposed strategy has not only improved classification metrics but it also enhanced the stock market returns, risks and risk to reward ratio of each ML model. Additionally, the results also revealed that how important it is to consider both classification as well as financial analysis to evaluate the performance of the ML model on stock market.

## Supporting information

S1 File Github file. The data and script has been uploaded to GitHub. It can be accessed using the following link: https://github.com/AzazHassankhan/Machine-Learning-based-Trading-Techniques/.
(IPYNB)
